# PhaseFIT: live-organoid phase-fluorescent image transformation via generative AI

**DOI:** 10.1038/s41377-023-01296-y

**Published:** 2023-12-14

**Authors:** Junhan Zhao, Xiyue Wang, Junyou Zhu, Chijioke Chukwudi, Andrew Finebaum, Jun Zhang, Sen Yang, Shijie He, Nima Saeidi

**Affiliations:** 1 Division of Gastrointestinal and Oncologic Surgery, Department of Surgery, Massachusetts General Hospital, Boston, MA 02114 USA; 2 Department of Surgery, Center for Engineering in Medicine and Surgery, Massachusetts General Hospital, Boston, MA 02114 USA; 3 Department of Biomedical Informatics, Harvard Medical School, Boston, MA 02115 USA; 4 Department of Biostatistics, Harvard T.H. Chan School of Public Health, Boston, MA 02115 USA; 5 College of Biomedical Engineering, Sichuan University, Chengdu, Sichuan 610065 China; 6 Shriners Hospital for Children—Boston, Boston, MA 02114 USA; 7 Tencent AI Lab, Shenzhen, Guangdong 518057 China; 8 Harvard Stem Cell Institute, Cambridge, MA 02138 USA

**Keywords:** Photonic devices, Imaging and sensing

## Abstract

Organoid models have provided a powerful platform for mechanistic investigations into fundamental biological processes involved in the development and function of organs. Despite the potential for image-based phenotypic quantification of organoids, their complex 3D structure, and the time-consuming and labor-intensive nature of immunofluorescent staining present significant challenges. In this work, we developed a virtual painting system, PhaseFIT (phase-fluorescent image transformation) utilizing customized and morphologically rich 2.5D intestinal organoids, which generate virtual fluorescent images for phenotypic quantification via accessible and low-cost organoid phase images. This system is driven by a novel segmentation-informed deep generative model that specializes in segmenting overlap and proximity between objects. The model enables an annotation-free digital transformation from phase-contrast to multi-channel fluorescent images. The virtual painting results of nuclei, secretory cell markers, and stem cells demonstrate that PhaseFIT outperforms the existing deep learning-based stain transformation models by generating fine-grained visual content. We further validated the efficiency and accuracy of PhaseFIT to quantify the impacts of three compounds on crypt formation, cell population, and cell stemness. PhaseFIT is the first deep learning-enabled virtual painting system focused on live organoids, enabling large-scale, informative, and efficient organoid phenotypic quantification. PhaseFIT would enable the use of organoids in high-throughput drug screening applications.

## Abstract (web summary)

PhaseFIT is a cutting-edge, segmentation-informed generative AI which effortlessly transforms one dimensional phase contrast images into rich, multi-channel fluorescent visuals for rapid, annotation-free phenotypic quantification, setting a new standard for high content drug screening applications.

## Graphical abstract

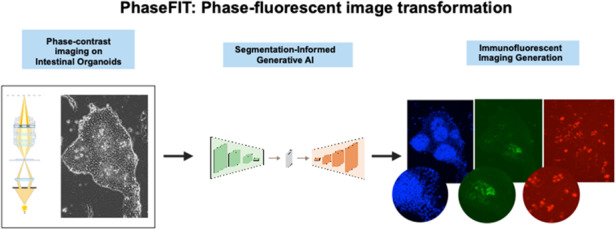

## Introduction

Phase-contrast imaging is a low-cost and readily available technique commonly employed to observe the spreading, growth, and morphology of cells in vitro. On the other hand, immunofluorescent (IF) imaging remains the gold standard for discerning high-contrast molecular specificity (e.g., to distinguish specific cell types). However, IF-based imaging is time-consuming, labor-intensive, and detrimental to cells, as it requires sample preparation and staining with dyes and typically only works for fixed, non-viable cells. Recently, quantitative phase imaging (QPI) has emerged as a powerful, label-free, and live cell-based approach to provide quantitative information about cell number, morphology, and cell cycles^1–3^. Moreover, deep neural networks (DNNs) have been trained to facilitate digital staining on phase images, such as, digital histological hematoxylin and eosin (H&E) staining and Masson’s trichrome staining on the phase images of tissue sections^4^, and digital fluorescent staining of specific proteins, such as Tau/Map2, on the phase images of neurons^5^. Yet, deep learning-enabled phase-contrast imaging analysis has not been utilized to quantify the phenotypes of organoid system, which contains high-dimensional and complex cellular composition with heterogeneous distributions.

An organoid is a mini organ in in vitro culture, which can mimic the diverse cell populations, complex anatomy, and physiological functions of in vivo organs. For example, same with the native intestinal epithelium, intestinal organoids exhibit distinguished crypt–villus structures wherein stem cells and Paneth cells reside in the crypt regions, and the absorptive cells (i.e., enterocytes), and the secretory cells (e.g., goblet cells and enteroendocrine cells) are in the villus regions^6,7^. Moreover, gut organoids have been used to model specific gastrointestinal diseases, such as colorectal carcinoma (CRC)^8,9^ and inflammatory bowel diseases (IBD)^10^. While organoid culture is a rapidly emerging platform with broad applications in both basic science and translational medicine, due to the intractable nature of the IF process for the complex 3D structures of the organoid system, the image-based phenotypic quantification of 3D organoids is usually limited to organoid number, size, and viability based on phase/brightfield imaging. Even though 2D and 2.5D organoid culture models that have recently been developed are more compatible with multiplex immunofluorescence (mpIF)^7,11,12^, mpIF is still an expensive and time-consuming process with large batch-to-batch heterogeneity. Thus, there is a significant need for the development of strategies for affordable, efficient, and informative organoid phenotypic quantification for large-scale applications, such as organoid-based drug screening.

Previous applications in cross-modality image transformations, such as phase-H&E virtual staining or H&E to mpIF translation, typically leverage deep generative adversarial networks and use one specific type of microscopic image as an input to generate various other staining types^13^. Recent investigations showed the feasibility of using deep learning to bridge quantitative phase imaging and fluorescent stains for understanding cell viability^14^. However, fine-grained transformation of complex and high-content organoid phase-contrast images into multiplex fluorescent staining images remains technically challenging, especially with no assistance of manual annotations. This is because DNNs are required to be trained for a high level of accuracy in simulating the complex optical relationship, unclear morphological boundaries, and high-content heterogeneities in organoid phase images while locating the implicit and even sparse protein expressions in the phase images to be able to generate the virtual fluorescent painting.

In the present study, we developed phase-fluorescent image transformation (PhaseFIT) for deep generative model-enabled virtual multiplex fluorescent painting on annotation-free phase-contrast live-organoid images. PhaseFIT is specially designed based on the characteristics of the image transformation task, taking into consideration the challenges posed by the complexities associated with a phase image (which is comprised of a broad spectrum of black & white intensity associated with different cell types and intracellular organelles) and highly heterogeneous composition of intestinal epithelial cells (which include several different cell types with different sizes and cellular densities). To overcome these challenges, PhaseFIT incorporates a segmentation algorithm to perform the image translation task with precision. To the best of our knowledge, this is the first algorithm that applies a segmentation-informed model to the phase-fluorescent image transformation task.

To enhance image generation, PhaseFIT incorporates channel-wise attention to focus on the most influential feature maps, as well as spatial-wise attention to suppress redundant feature regions. These design choices ensure that PhaseFIT effectively captures and utilizes the critical information present in the images for improved cross-modality image-to-image translation. This segmentation-informed model reaches nearly real-time inference for transforming images from live organoid phase images to multiplex fluorescent staining. Moreover, the proposed algorithm outperforms the state-of-the-art generative adversarial networks-based stain transformation methods when generating virtual painting from heterogeneous and artifact-lean organoid phase images. To validate the sensitivity and usefulness of PhaseFIT, we quantified the effects of three compounds on the phenotypes of the treated organoids, which exhibited that PhaseFIT is a potent tool for image-based organoid phenotypic quantification in biomedical and biopharmaceutical studies.

## Results

### PhaseFIT workflow

**Fig. 1:**
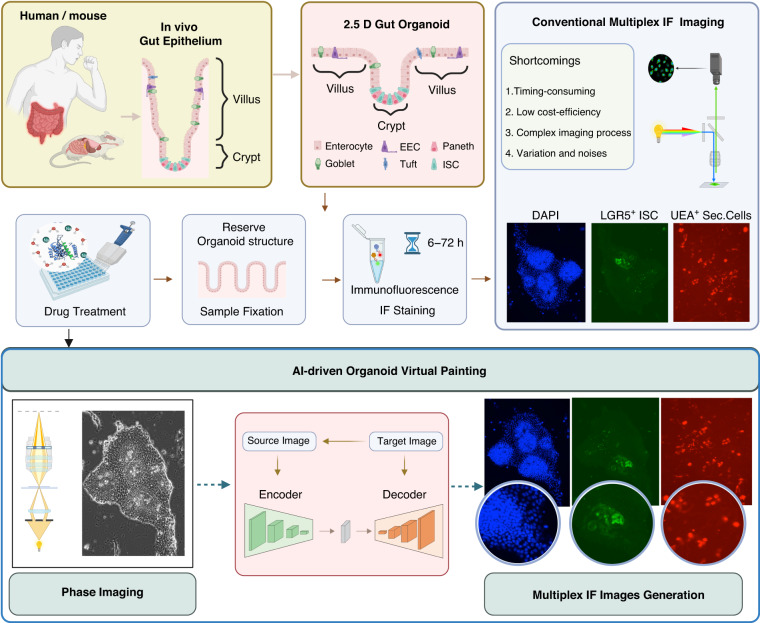
Workflow of live-organoid phase-fluorescent image transformation (PhaseFIT). The mouse/human-derived 2.5D organoids recapitulate the features of in vivo gut epithelium (such as the crypt-villus structure and the diverse cell populations) and are compatible with high-throughput screening (HTS). The conventional image-based phenotypic analysis exhibits four shortcomings, including time-consuming immunofluorescent staining, high cost for antibodies, complex system for multiplex image acquisition, and variations caused by staining and imaging between different benches. To overcome those shortcomings, PhaseFIT can transform the live-organoid phase images to generate multiplex fluorescent images using AI-driven virtual painting

We developed a virtual painting system, PhaseFIT to quantify the phenotypes of 2.5D intestinal organoids (Fig. 1) from the phase images, such as the size of crypt and villus regions, expression of stem cell markers, and the proportions of different cell populations. The 2.5D organoid model that we recently developed^7^ not only accurately mimics the key features of in vivo gut epithelium (such as the crypt-villus structure and the diverse cell populations), but it also bypasses the tedious culture and staining protocols that are required for the conventional 3D organoids. Moreover, the 2.5D organoid model is readily amenable to being integrated into high-throughput screening platforms that would be necessary for large-scale drug testing and validation. However, the standard fluorescence imaging-based phenotypic analysis is not conducive to high-throughput systems owing to some of its limitations, including the time-consuming staining process, high cost of antibodies, complex system for multiplex imaging acquisition, and artificial variations in both staining and imaging across different batches (Fig. 1). PhaseFIT transforms the phase images of live organoids to generate multiplex fluorescent images using an AI-driven virtual painting algorithm. By bypassing the conventional imaging acquisition, PhaseFIT can report organoid phenotypes based on the live-organoid phase images in a real-time manner (Fig. 1).

**Fig. 2:**
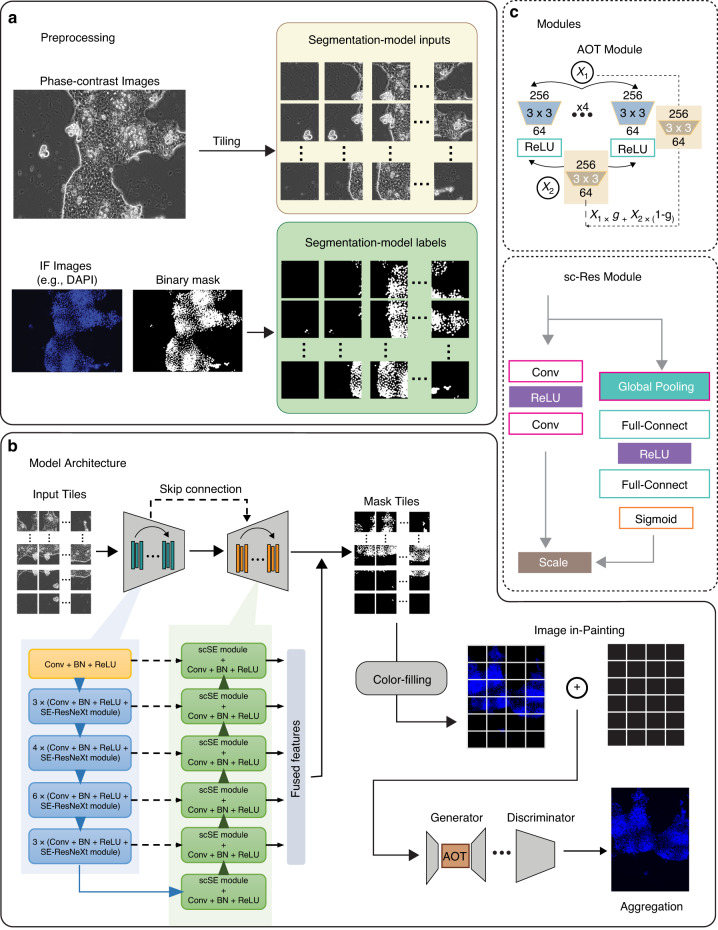
Segmentation-informed deep generative model architecture. **a** Image pre-processing leverages multi-instance learning by splitting into patches and converting them into binary images. **b** The model architecture consists of a segmentation-informed framework with channel-wise and spatial-wise attention modules. **c** The AOT module and sc-Res module are employed to smooth tile concatenation and enhance feature selection, respectively, ensuring accurate and detailed segmentation

The proposed segmentation-informed generative model (Fig. 2a) was trained on pairs of fluorescent and phase-contrast images of the organoids and was designed for overcoming the challenges of sparse stains and artifacts. To maintain the contextual granularity and objective sensitivity of virtual painting, we incorporated the concept of splitting each image into tiles with 50% overlapping between each pair of adjacent tiles (Fig. 2b). These tiles were used as separate inputs to generate corresponding segmentation results. These predicted segmentation results were then aggregated into an image and a color fill operation was performed to obtain the transformed image. We adopted an aggregated contextual transformation (AOT) block optimized for context reasoning in high-resolution image inpainting^15^. AOT blocks employ a split-transformation-merge strategy, allowing the generator to predict each output pixel. AOT blocks do not introduce additional parameters or computational costs. The AOT block’s design enables it to capture rich patterns of interest by learning aggregated contextual transformations with various dilation rates, making it highly effective for context reasoning in image inpainting (Fig. 2c).

### Virtual painting performance of PhaseFIT for generating nuclei, stem cells, and secretory cells

**Fig. 3:**
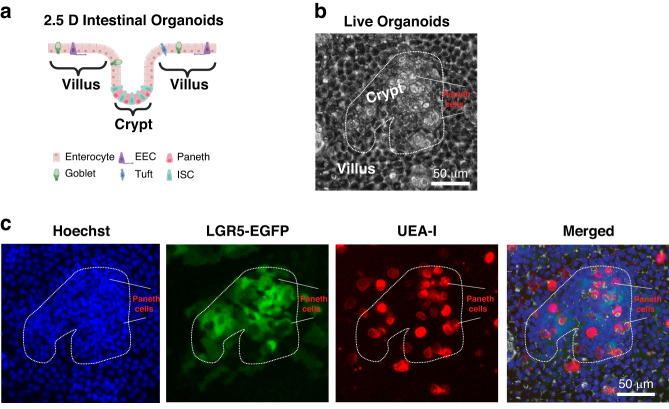
Staining 2.5D gut organoids with Hoechst, LGR5-EGFP and UEA-I for phenotypic profiling. **a** Illustration for the 2.5D gut organoids including the crypt-villus structure as well as the distribution of different cell populations. ISC, intestinal stem cells; EEC enteroendocrine cells. **b** A representative live organoid phase image with visible features for crypt vs. villus regions and Paneth cells. P Paneth cells. **c** Live imaging for Hoechst nucleus stain, LGR5-EGFP^+^ ISCs, and UEA-I^+^ secretory populations, corresponding to the live phase image in panel (**b**)

The signals from the two immunofluorescence dyes (i.e., Hoechst and UEA-I) and the protein LGR5-EGFP were chosen to exhibit the high-physiologically relevant features of the 2.5D organoids (Fig. 3a)^7^. Hoechst nucleus staining with the super dense nucleus distribution in the crypt regions (i.e., stem cell niche) can efficiently identify the crypt-villus structure (Fig. 3b and c). Stem cells and differentiated cells are located in the crypt and the villus regions, respectively (Fig. 3a and b). Thus, we can use the size and number of the crypts or villus surface areas calculated based on nucleus staining as metrics to quantify the dynamics of the crypt-villus structure. The 2.5D organoids encompass the diverse cell populations found in the in vivo gut epithelium, including intestinal stem cells (ISCs), absorptive enterocytes, and secretory cells (such as Paneth cells, goblet cells, and enteroendocrine cells, Fig. 3a)^7^. LGR5-EGFP and UEA-I, a fluorescent lectin stain, are respectively used to identify ISCs and the secretory cell populations (Fig. 3c). UEA-I^+^ cells in the crypt regions are Paneth cells^16^, and those in the villus regions are other types of secretory cells^17,18^ (Fig. 3c). We have previously demonstrated that different gastrointestinal pathological conditions can be accurately captured in the 2.5D organoid system by variations in the villus-crypt structure and the proportions of different cell populations. For example, decreased crypt size and loss of ISC population are observed during inflammatory bowel diseases (IBD)^7^, and conversely, increased crypt size and gain of ISC number indicate tumorigenesis^19^.

**Fig. 4:**
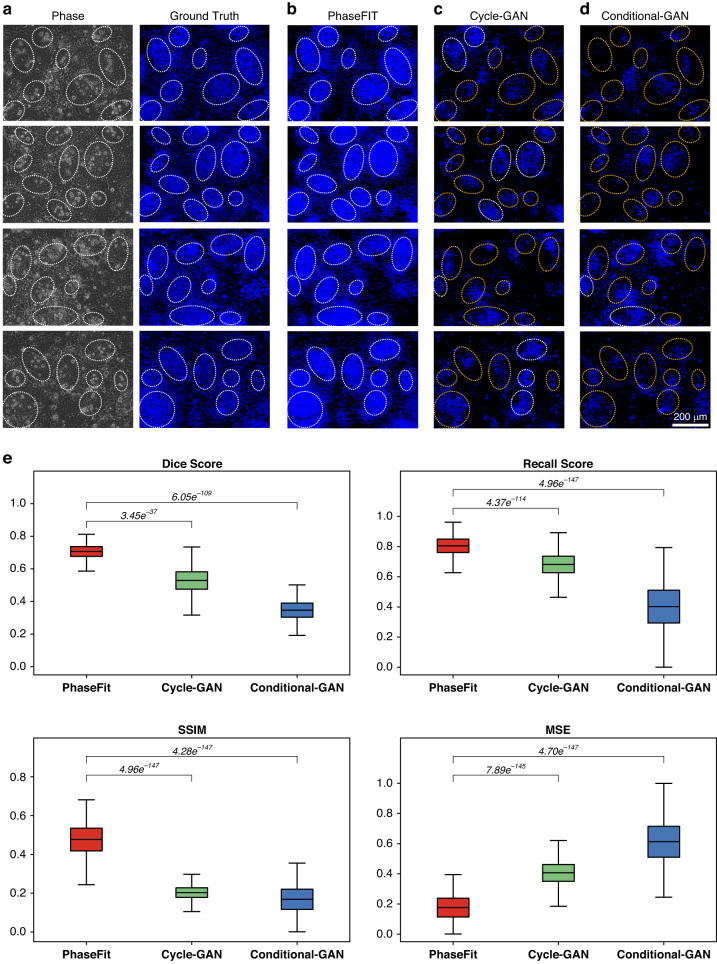
Comparisons of PhaseFIT against state-of-the-art GAN methods for virtual nucleus painting. Compared to the ground truth from real nucleus DAPI staining (**a**), the performance of PhaseFIT (**b**) is better than the GAN methods (**c** and **d**) with respect to identifying the crypt-villus structure. The white dashed lines indicate that the virtual painting successfully captured the crypt regions. Otherwise, the yellow dashed lines indicate that the crypt regions were missed in the virtual painting. **e** The performance of these three algorithms was compared by quantifying the similarity between their vial painting results and the ground truth images using four different methods (Dice, Recall, SSIM, and MSE). PhaseFIT showed a Dice score of 0.71 ± 0.044, a recall score of 0.80 ± 0.065, a structural similarity index measure (SSIM) of 0.47 ± 0.086 and a pixel-wise MSE of 0.17 ± 0.091, outperforming Cycle-GAN (0.53 ± 0.083, 0.68 ± 0.085, 0.20 ± 0.038, and 0.40 ± 0.087), and Conditional-GAN (0.34 ± 0.063, 0.39 ± 0.158, 0.16 ± 0.075, and 0.60 ± 0.149). *p* < 0.001 (*n* = 889, paired Wilcoxon rank-sum test)

**Fig. 5:**
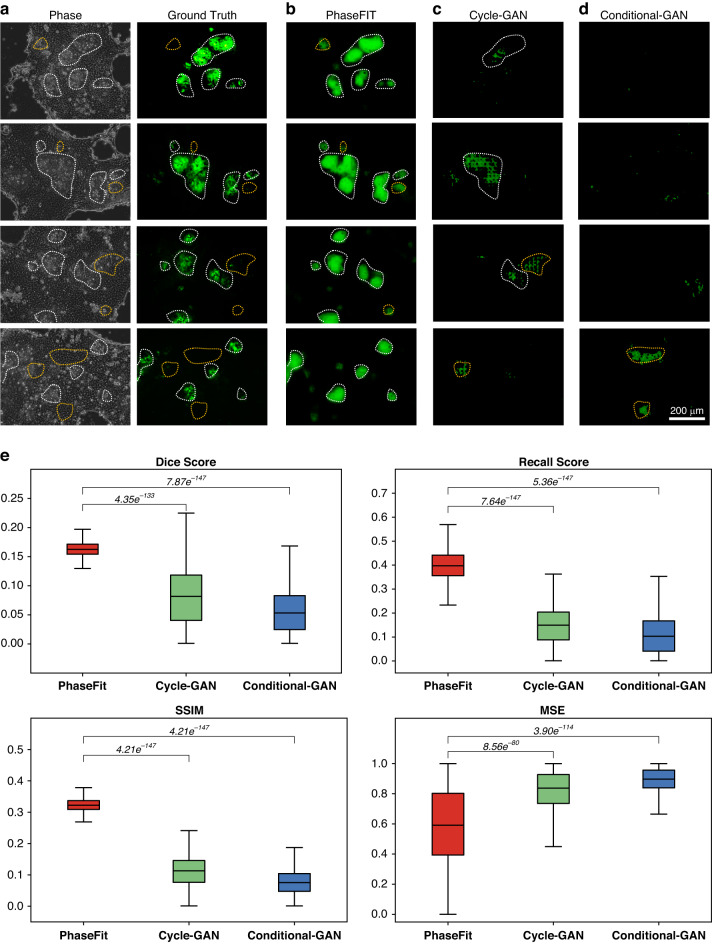
Comparisons of PhaseFIT against GAN methods for virtual LGR5 painting. LGR-5 is the marker for intestinal stem cells located in crypts. Compared to the ground truth from LGR5-EGFP^+^ cells (**a**), the performance of PhaseFIT (**b**) is much better than the GAN methods (**c** and **d**) which almost failed to identify the LGR5^+^ cells. The white dashed lines indicate that the virtual painting successfully captured the LGR5-EGFP signals. Otherwise, the yellow dashed lines indicate that the virtual painting generated false LGR5 signals. **e** PhaseFIT dominated the task with a Dice score of 0.16 ± 0.013, a recall score of 0.40 ± 0.063, an SSIM of 0.32 ± 0.020, and a MSE of 0.58 ± 0.300, in comparison to Cycle-GAN (0.08 ± 0.058, 0.15 ± 0.087, 0.11 ± 0.052, and 0.84 ± 0.143) and Conditional-GAN (0.05 ± 0.043, 0.11 ± 0.093, 0.90 ± 0.086, and 0.95 ± 0.039). *p* < 0.001 (*n* = 889, paired Wilcoxon rank-sum test). 4.12e^−^^147^ is the smallest value

**Fig. 6:**
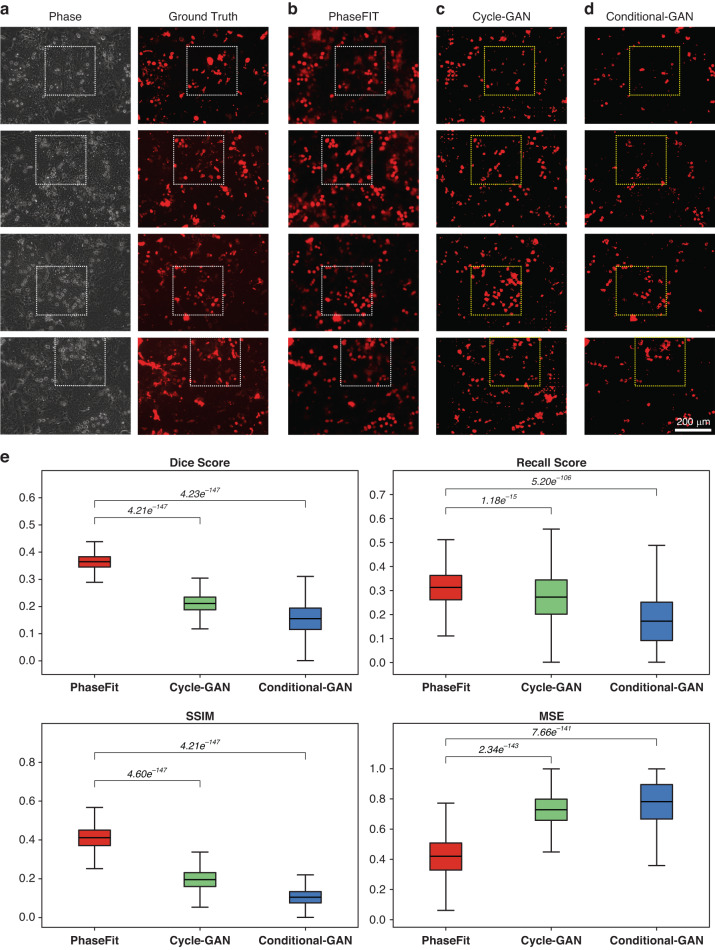
Comparisons of PhaseFIT against GAN methods for virtual UEA-I painting. UEA-I stains the secretory cells, such as Paneth cells and goblet cells. Compared to the ground truth from real UEA-I staining (**a**), the performance of PhaseFIT (**b**) is better than the GAN methods (**c** and **d**). For example, the GAN methods, especially Conditional-GAN, frequently missed the UEA-I^+^ dot-like signals in the dashed boxes (**e**) PhaseFIT model exhibited a superior Dice score of 0.36 ± 0.028, a recall score of 0.31 ± 0.077, a SSIM of 0.41 ± 0.061, and an MSE of 0.41 ± 0.136. This was marginally higher compared to Cycle-GAN’s 0.21 ± 0.036, 0.28 ± 0.110, 0.20 ± 0.055, and 0.73 ± 0.107; Conditional-GAN’s 0.16 ± 0.057, 0.18 ± 0.116, 0.11 ± 0.042, and 0.79 ± 0.165. *p* < 0.001 (*n* = 889, paired Wilcoxon rank-sum test). 4.12e^−^^147^ is the smallest value

We trained the PhaseFIT with ground truth images from the real Hoechst and UEA-I staining (Figs. 4 and 5) and from the LGR5-EGFP (Fig. 6) (see the “Materials and methods” section). We compared PhaseFIT’s performance with two previously reported generative adversarial network (GAN)-based methods that demonstrated promising image translation performances. More specifically, Conditional-GAN was applied in DeepLIIF for deconvoluting H&E or IHC images of tissue section samples into multiplex fluorescent stains with human-annotated ground truth masks and supervised segmentation^20,21^. By contrast, CycleGAN improved the robustness in unsupervised learning and cross-domain translation for transforming H&E staining images into special stains^22–26^. We trained all three models and tested them using an identical dataset (see the “Materials and methods” section). Four quantitative measures of Dice, pixel-wise Recall (sensitivity) scores, structural similarity index (SSIM), and mean squared error^27–29^ were tested for comparing the qualities of the generated images on both local pixel level and global cell morphological level (see the “Materials and methods” section).

Compared to the ground truth from real Hoechst nucleus staining, the virtual nucleus staining images generated by PhaseFIT showed superiority compared to the GAN methods with respect to identifying the crypt-villus structure. All the crypt regions identified from ground truth images (white dashed lines in Fig. 4a) were perfectly captured by PhaseFIT (Fig. 4b). The Cycle-GAN model, however, missed multiple small crypt regions per field of view (indicated by the yellow dashed lines in Fig. 4c). The Conditional-GAN failed to reconstruct the crypt regions (shown by the red dashed lines in Fig. 4d). Notably, PhaseFIT provides the best contrast for qualitatively identifying the crypt regions among the three models. PhaseFIT achieved high scores in all metrics, significantly outperforming both Conditional-GAN and Cycle-GAN, and demonstrating the superior performance of PhaseFIT in generating virtual nucleus stain (Fig. 4e).

The accuracy of Lgr5 virtual painting depends on the ability of a generative model to detect the outlines of crypts in phase-contrast images during the training process. This new approach differs from GAN-based methods by overlaying morphological patterns for the region-of-interest (ROI) between phase-fluorescent pairs using segmentation, delivering a more precise and efficient virtual painting compared to the other two GAN models. PhaseFIT accurately recapitulated the LGR5 signals in the ground truth images (Fig. 5a and b). However, the two GAN models (Fig. 5c and d) only captured the LGR5 signals in very sparse positions (shown by the white dashed lines in Fig. 5c and d) and even produced false LGR5 signals (indicated by the yellow dashed lines in Fig. 5c and d). Thus, the GAN models are unable to generate the virtual LGR5-EGFP stain. The Dice, Recall and SSIM scores confirmed that compared to the two GAN methods, PhaseFIT can accurately generate the LGR5 stain, which better recapitulates the real LGR5-EGFP signals in the ground truth images (Fig. 5e).

PhaseFIT successfully captured the morphological features of UEA-I^+^ cells in the live phase images (Fig. 6a). Compared to the PhaseFIT (Fig. 6b), the other two GAN models, particularly the Conditional-GAN model, did not efficiently generate the UEA-I staining and frequently missed real signals (as shown in the dashed boxes in Fig. 6c and d). Consistently, all four measures suggest that PhaseFIT is superior to Cycle-GAN and Conditional-GAN. In summary, the segmentation-informed PhaseFIT significantly outperforms the other GAN models and can be applied to generating all three virtual stains.

### PhaseFIT-based drug screening

**Fig. 7:**
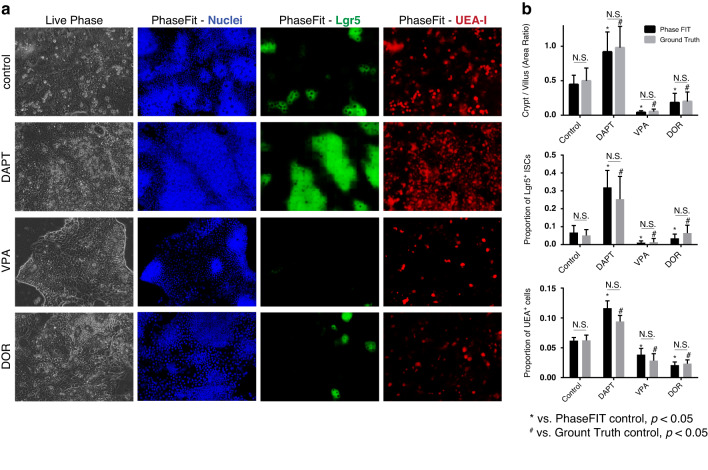
Quantification of drug impacts on the organoid phenotypes via PhaseFIT. **a** PhaseFIT generated virtual staining of nuclei, LGR5, and UEA-I for four groups, control, DAPT, VPA, and DOR. **b** DAPT significantly increased the area ratio between crypt and villus regions, and the proportions of LGR5^+^ ISCs and UEA-I^+^ secretory cells in total cell number. In contrast, VPA and DOR significantly decreased them. The quantification from PhaseFIT virtual painting was consistent with the ground truth staining images. * vs. PhaseFIT control and # vs. ground truth control, *p* < 0.05 (*n* = 18, Student’s *t*-test)

As a proof-of-concept for the suitability of PhaseFIT for drug screening applications, we applied it to assess the impact of three previously described chemical compounds on organoid phenotypes, DAPT, Valproic acid (VPA), and Dorsomorphin (DOR). These compounds have been used to manipulate the ISC differentiation in previous studies: DAPT induces secretory cell differentiation by inhibiting the Notch signaling pathway^30^. In contrast, VPA promotes enterocyte differentiation by activating the Notch signaling pathway^31^. DOR increases villus size by inhibiting the bone morphogenetic protein (BMP) signaling pathway^32^. After administering these compounds, we collected live organoid phase images as input for the PhaseFIT workflow to generate virtual multiplex images for DAPI, LGR5, and UEA-I (Fig. 1). The virtual PhaseFIT painting images revealed that, compared to the vehicle control group, DAPT expanded the crypt region area and increased the population of Lgr5^+^ ISCs and UEA-I^+^ secretory cells. In contrast, both VPA and DOR inhibited crypt formation and decreased the populations of Lgr5^+^ ISCs and UEA-I^+^ secretory cells (Fig. 7a). We confirmed these phenotypes through the quantification of the area ratio between crypt and villus regions and the proportion of LGR5^+^ ISCs and UEA-I^+^ secretory cells (see the “Materials and methods” section and Fig. 7b). We observed no statistically significant differences in the cell population quantification from virtual painting images generated by PhaseFIT and those from the ground truth (real painting) images (Fig. 7b), confirming the accuracy of PhaseFIT virtual painting in analyzing organoid phenotypes.

## Discussion

As an emerging technique, in vitro organoid models, which faithfully serve as surrogates of in vivo organs, are the next generation of in vitro culture systems quickly replacing the conventional cell culture models. Organoids also provide a complementary alternative to animals as in vitro models of disease and for toxicological testing. Considering the labor-intensive and time-consuming nature of acquiring fluorescent images, extracting cell-level information using the standard phase contrast images would significantly broaden the large-scale applications of organoids.

During the past 10 years, deep learning models have been trained to recognize biological and clinical images for biomedical and therapeutic applications^33,34^. Specifically, the GAN-based methods, such as, Conditional-GAN^20,21^ and Cycle-GAN^22–26^, are used for transforming between different stains, such as H&E, IHC, or phase images of tissue section samples. However, transferring phase-contrast images to fluorescent images using deep learning techniques presents several challenges that GAN-based methods cannot easily overcome. That includes the sensitivity to imperfections in the optical path, which may be a result of misaligned mirrors or lenses and consequently result in blurred images. Analyzing organoids using phase-contrastive imaging are much more challenging than cells, even with expert annotations. Meaningful unbiased analysis, interpretation, and manipulation of these highly complex images require even more complex levels of computation, and this is where PhaseFIT excels.

PhaseFIT is specifically designed for live-organoid phenotypic profiling via generating informative virtual fluorescent stains based on phase images of live organoids. PhaseFIT leverages the advancement of segmentation by identifying and separating cells and structures within a phase-contrast image, especially when there is an overlap or close proximity between objects. We applied PhaseFIT on 2.5D gut organoids^7^ and trained it to generate virtual DAPI nuclear staining, LGR5-EGFP signals, and UEA-I staining. These multiplex fluorescent images provided comprehensive interpretable phenotypic profiling, including distinguishing crypt-villus structure based on nuclear distribution and quantifying the proportion of the LGR5^+^ stem cells and UEA-I^+^ secretory cells. These phenotypes are highly physiologically relevant. The changes in crypt structure and stem cell populations are involved in the development of both IBD and cancer^7,19^. We confirmed that PhaseFIT significantly outperformed the two previously developed GAN-based methods in generating fluorescent representations of the crypt regions and stem cell populations. Our primary objective is to identify the overall regions that are populated by the cells and accentuate contrasts between different cell types. This aids in facilitating scientific exploration and analysis and does not necessitate the generation of realistic nucleus contours. It is particularly challenging given the complexities associated with a phase image, which is comprised of a broad spectrum of black & white intensity associated with different cell types and intracellular organelles and highly heterogeneous composition of intestinal epithelial cells, which include several different cell types with different sizes and cellular densities. Indeed, even an expert eye is unable to distinguish between the different cell populations solely from a phase image. It is notable that despite these challenges, PhaseFIT, as underscored by both qualitative and quantitative measures, exhibits at least a two-fold improvement in sensitivity compared to the existing state-of-the-art methodologies. Of note, PhaseFIT is not limited to the three virtual stains that were developed here. Following the same workflow, PhaseFIT can be expanded to generate virtual painting for other signals (e.g., for other specific cell sub-populations) once these signals can be visualized to train the model.

PhaseFIT has been specifically designed to address the challenges that are associated with background noise in fluorescent images. The segmentation-informed module included in PhaseFIT helps to overcome interference and accurately distinguishes between genuine signals and background noise. Additionally, PhaseFIT employs an attention-based approach to tackle the challenges related to staining variability between or within cells. This approach is particularly beneficial when working with multi-channel stains, as it ensures accurate alignment and improves overall accuracy of virtual painting.

Label-free PhaseFIT provides an efficient, accurate, and unbiased workflow for quantifying organoid phenotypes by bypassing the time-consuming, expensive, and complex processes of fixation, staining, and multiplex imaging required for fluorescence imaging. In addition, the virtual painting generated from PhaseFIT is based on the live organoid phase images, which preserve the organoid morphology in live status without any artificial perturbation and can be processed in real-time (necessary for assessing time-dependent responses).

As a proof-of-concept in the application of drug screening, we demonstrated that PhaseFIT can reliably quantify the impacts of three chemical compounds on the organoid phenotypes, underscored by both qualitative and quantitative measures, exemplifies the successful implementation of PhaseFIT and attests to its ability to fulfill the requisite requirements. Specifically, DAPT increased the crypt size and increased the populations of ISCs and secretory cells. VPA and DOR showed impacts that were opposite to DAPT. These phenotypes were confirmed with the ground-truth images and are consistent with previous reports^30–32^.

After the FDA announced the removal of the mandatory animal testing requirement before commencing human trials for all drugs, we envision the future of in vitro research and acknowledge the immense scalability of organoid cultures. Herein, we present PhaseFIT, the first generative AI system designed to facilitate the transition from phase-contrast images to multipixel Immunofluorescence (IF) staining images. This development addresses the rapidly growing need for organoids in high-throughput analyses and enhances the utility of the extensive repositories of phase-contrast images available in the scientific community. The introduction of PhaseFIT could amplify and accelerate the application of organoids in the domain of drug discovery. Hence, it is poised to significantly propel advancement in these crucial research fields.

## Materials and methods

### Animals

We used Lgr5-EGFP-IRES-CreERT reporter mice^35^ for isolation of the intestinal crypts (which contain the stem cells and their niche, Paneth cells). In these mice, the LGR5 knock-in allele has mosaic expression in the intestine and expresses a fusion protein of EGFP and CreERT2. Thus, this model allows for visualization of ISCs and progenitor cells based on GFP expression. Animal studies were conducted in accordance with the National Institutes of Health “Guide for the Care and Use of Laboratory Animals” (NIH Publication No. 85-23, revised 1996) and approved by the Institutional Animal Care and Use Committees (IACUC) of the Massachusetts General Hospital.

### Harvest of crypts

The proximal 12–15 cm small intestines were collected from 10 to 14 weeks old mice from either sex. The intestinal lumen was opened longitudinally. The mucous was removed using the blunt side of the blades. Then, the intestine was washed with ice-cold PBS without calcium and magnesium (Corning, 21-040), and cut into 5 mm–1 cm fragments, and placed into 50 ml conical tubes that were filled with ice-cold 50 ml of PBS/EDTA (10 mM, Thermo Fisher, 15575020). The fragments were incubated and shaken on ice for 40 min and washed with ice-cold 50 ml of PBS. Then, the fragments were vigorously shaken in 25 ml PBS and filtered twice through a 70 μm mesh (BD Falcon) into a 50 ml conical tube to remove the villi and tissue pieces. The crypts were mainly in the suspension which were centrifuged for 5 min at 100×*g*. The crypt pellets collected here were then used for seeding on the hydrogel.

### In vitro culture of 2.5D organoids

The crypt pellets were suspended in the seeding media and counted using a cytometer to control the crypt density as 10,000/ml. 30 μl crypt suspension was added to a 96-well plate coated with a 2.4 kPa polyacrylamide (PA) hydrogel matrix. PA hydrogel was fabricated as previously described^36^. Briefly, the recipe for different Young’s modulus was 7.5% acrylamide and 0.034% bisacrylamide for 2.4 kPa. 0.1% ammonia persulfate (sigma, 09913) and 0.05% TEMED (Bio-Rad, 1610800) were added to start the polymerization process. The polymerization required 40 min to 1 h. Then, sulfo-SANPAH (Proteochem, C1111) was used to activate the gel surface under a 15 W 365 nm UV (VWR, 95-0042-07/36575-050) for 10 min. After the activation, 0.1 mg/ml type I collagen (Advanced biomatrix, 5022) was added onto the gel overnight to covalently attach to the gel surface for the organoid culture.

Four hours after seeding the crypts, the floating cells/clusters were washed with PBS (Corning, 21-040-cv). 0.3 ml ENR (EGF, Noggin, and R-spondin) media/well was added and changed every other day. To make the ENR media, advanced DMEM/F12 (Gibco, 12634-028) was supplemented with 50 ng/ml EGF (Peprotech, 315-09), 100 ng/ml Noggin (Peprotech, 250-38), 10% R-spondin conditional media (iLab in Harvard digestive center), 1% Glutamax (Gibco, 35050-061), 1% HEPES (Gibco, 15630-080), 0.2% Primocin (Invivogen, ant-pm-2), 0.2% Normocin (Invivogen, ant-nr-2), 1% B27 (Gibco, 12587010), 0.5% N2 (Gibco, 17502-048), and 1.25 mM N-Acetyl-Cystein (Sigma, A8199). To make the seeding media, the ENR media was supplemented with 3 μM Chir-99021 (Selleckchem, S1263) and 10 μM Y-27632 (Sigma, Y0503). 10 µM DAPT in DMSO, 1 mM Valproic acid (Sigma, PHR1061), and 5 µM Dorsomorphin (Sigma, P5499) in DMSO were supplied as needed.

#### Data acquisition and analysis

After 6 days of culture, the living cells were directly stained with Hoechst 33342 (Thermofisher, 62249, 1 µM) and Ulex Europaeus Agglutinin I (UEA-I, DyLight™ 594, DL-1067-1, 1:500) for 30 min. Immediately after the staining, the living cells were imaged using ×20 objective of EVOS M5000 with the phase channel and the DAPI, GFP, and TexRed light cubes (for Hoechst, Lgr5, and UEA-I, respectively). For training purposes, 50 wells (10 per mouse) were prepared for each channel, and 889 images (10 per well) were acquired. For the drug testing experiments, six wells (three per mouse) were prepared, and 18 images (three per well) were acquired for each channel. Crypt regions are featured with more dense nuclei than villus regions (Fig. 4). Therefore, based on the nuclear staining the areas of villus and crypt regions were quantified using Image-J to calculate the area ratio between crypt and villus regions (Fig. 7b). In addition, to quantify the proportion of LGR5^+^ ISCs, and UEA^+^ secretory cells (Fig. 7b), Image-J was used to count the numbers of total cells, LGR5^+^ ISCs, and UEA^+^ secretory cells, respectively, based on the staining of nuclei, LGR5 and UEA-I.

#### Image pre-processing

To maintain precise segmentation, the original images (source) and the corresponding masks (target) with a size of 1536*1024 pixels are cropped into smaller patches (256*256 pixels) with 50% overlapping (Fig. 2). These obtained patches are independently used as the new source and target images for the image segmentation task. Since the segmentation model requires binary images as the supervision signal for network training, these color target images are converted into binary images by a simple thresholding technique. These thresholds are determined by the background pixel values of the three types of color images.

#### Baseline stain transformation deep learning models

We used two state-of-the-art stain transformation (virtual staining) deep generative models for comparisons with the proposed method. The model families can be represented as conditional-GAN-based DeepLIIF^20^ and Cycle-GAN-based PhaseStain^26^. Both frameworks are common image-generation methods that can be applied in various applications. We used the original public codes and the experimental setups as the authors listed.

#### Segmentation-informed deep generative network

Although deep generative networks have shown remarkable success in generating high-quality data that is difficult to distinguish from real data, one of their key challenges is the ability to capture the complex structure and patterns of the data in a compact and efficient manner. Current deep generative networks can deal with the tasks of translating one possible representation of a scene into another. If the given scene contains additional noise information, the network could generate undesired output^37^. To address this issue, we designed a novel deep generative network to output three individual modalities, DAPI (in blue), Lgr5 (in green), and UEA-I (in red), given a set of phase-contrast images (in grayscale) as inputs. As seen in Fig. 2, the representations of the source inputs and target outputs are only partially similar in texture and morphology, thus this task cannot be simply regarded as an image-to-image translation problem that has achieved great success in the field of natural images based on generative models (e.g., Cycle-GAN^22^). Additionally, the challenges of the noises of artifacts and implicitly sparse distributions of the stains, such as UEA-I, need to be considered. Therefore, we deployed a semantic segmentation strategy to operate the image-to-image generation task, which helps localize these key cell-related regions and generate the targets precisely.

This segmentation model is constructed using an improved U-Net framework by adding channel-wise attention to focus on these most contributing feature maps and spatial-wise attention to suppress redundant feature regions (e.g., excessive background). Specifically, the encoder part uses the SE-ResNeXt50, which is obtained by incorporating the SE module into the ResNeXt50 architecture, enabling the network to learn adaptive channel-wise feature dependencies. The SE module is inserted between two convolutional layers in each ResNeXt block. ResNeXt50 is an extension of the ResNet architecture that utilizes grouped convolutions to enhance its representational capacity. SE-ResNeXt50 comprises five down-sampling stages to obtain hierarchical features, where each stage includes three convolution operations, three batch normalization modules, two ReLU operations, and a squeeze-and-excitation (SE) module^38^. The SE model has two main components: the squeeze operation and the excitation operation. The squeeze operation applies a global pooling layer to the input feature map, reducing the spatial dimensions to a single value per channel. This captures channel-wise statistics and provides a global view of the feature distribution. In the excitation operation, the output of the squeeze operation passes through two fully connected layers. These layers learn a set of channel-wise weighting coefficients that rescale the original feature map. This amplifies the saliency of the most informative channels while suppressing the less relevant ones.

The encoder generates high-level features and increases the feature diversity by reducing the spatial size of the feature matrix and increasing the number of feature channels/maps. These feature maps can be regarded as different views obtained from different perspectives of the image representation. The SE module is used to impose more weight on the feature maps that contribute the most. The decoder part consists of five up-sampling stages to recover image resolution, where each network block includes two sequential convolution, batch normalization, and ReLU operations, as well as a concurrent spatial and channel squeeze & excitation (scSE) module^38^. The scSE module extends the SE module by incorporating both channel-wise and spatial-wise feature dependencies using two parallel branches. The first branch performs the original SE module’s channel-wise squeeze and excitation operations, while the second branch performs spatial squeeze and excitation operations. In the spatial squeeze operation, a global average pooling layer is applied along the channel axis to reduce the channel-wise feature maps to a single spatial value. This captures the spatial-wise statistics of the feature map, providing a global view of the spatial distribution. In the spatial excitation operation, weights are learned for each spatial location by applying two fully connected layers to the squeezed feature descriptor. These weights are then used to rescale the original feature map, emphasizing important spatial features and suppressing less important ones.

At each decoder stage, it combines these features at the corresponding encoder level using skip connection (feature concatenation). Then, the features eventually used for prediction are concatenated from each decoder stage to form the fused features as shown in Fig. 2. It is seen that the number of feature maps in the decoder part is much larger than that in the encoder part.

Therefore, a feature map selection strategy is also necessary. In addition, the goal of the decoder is to gradually increase the feature size to obtain the final segmented image (target image). Thus, feature selection in the spatial view is also an important strategy. Based on the above considerations, we use the scSE module to combine channel-wise and spatial-wise concerns to focus on these regions with distinct appearances, which helps better segment the details.

The segmentation loss function combines the focal loss (1) and Dice loss (2), which have been shown to better handle data imbalance problems^39^. The focal loss applies larger weights to these hard-to-distinguish samples and conversely, smaller weights to these easy-to-distinguish samples. The Dice loss is used to measure the degree of overlap between two sets/regions. The two loss functions are calculated as follows:

1 $${L}_{{\rm{{FL}}}}=-{\sum }_{i}\left[{\left(1-\hat{{y}_{i}}\right)}^{{\rm{\gamma }}}{y}_{i}\log \hat{{y}_{i}}+{\left(\hat{{y}_{i}}\right)}^{{\rm{\gamma }}}\left(1-{y}_{i}\right)\log \left(1-\hat{{y}_{i}}\right)\right]$$

2 $${L}_{{\rm {{DICE}}}}=-\frac{2{\sum }_{i}^{N}{y}_{i}\hat{{y}_{i}}}{{\sum }_{i}^{N}{y}_{i}^{2}+{\sum }_{i}^{N}\hat{{y}_{i}^{2}}}$$

For the *i*th pixels in the training image, *y*_*i*_ represents its ground truth label and $\hat{{y}_{i}}$ denotes the predicted probability for the category with label 1. ${\rm{\gamma }}$ > 0 is a hyperparameter (focusing parameter), which is determined using an ablation experiment.

The model predicts the probability for each pixel of the tile image. These predicted tiles are then merged to generate segmented images. To obtain the target color images (DAPI nuclear staining, LGR5-EGFP signals, and UEA-I staining), the segmented images are in-painted by multiplying each pixel probability by 255. After the model outputs the inferences on each tile image, a context-aggregated image inpainting module is applied to smooth the tile concatenation using a binary mask, where non-zero pixels correspond to the division line between each tile pair. We use the aggregated contextual transformation (AOT) block to handle the spatial merging process^15^.

#### Experimental setups

All experiments in this study were constructed using a 5-fold cross-validation strategy, where four folds of the data were used for model training and one held-out fold was reserved for validation purposes. PhaseFIT model was trained using the Adam optimizer with an initial learning rate of 0.0003. The learning rate was then reduced by a factor of 10 at the 10th and 20th epochs. To increase sample diversity and mitigate overfitting, a data augmentation strategy was implemented. This strategy included random cropping, horizontal flip, vertical flip, random scaling, brightness change, contrast change, and Gauss noise change. Furthermore, a dropout layer with a probability of 0.5 was incorporated into the model architecture. The mini-batch size and the number of epochs were set to 24 and 30, respectively. All experiments were implemented in PyTorch and performed on a workstation equipped with four 32 GB NVIDIA Tesla v100 GPU cards. It takes <1 s mm^−1^ with a GPU to complete the virtual painting on a 96-well assay.

The Cycle-GAN and Conditional GAN (cGAN) used in this study as a comparison, were trained using their official implementations. Both the generator and discriminator in the cGAN and Cycle-GAN models were trained using the Adam optimizer with an initial learning rate of 0.0002 for a total of 200 epochs. The mini-batch size is set as 32. Unlike the PhaseFIT model, the GAN model takes the entire images as inputs (without clipping) to ensure better global image translation. To achieve this, we filled the input images to make them square by using the edge pixels. These filled edges were subsequently removed in the final prediction results.

#### Comparison with GAN-based models, and evaluation metrics

To create a fair comparison, we adopted cross-validation and partitioned the dataset into five folds. In each repetition, four folds were used for training PhaseFIT and the other two GAN models, and one fold served as the held-out test set that was never seen by the models. Four quantitative measures i.e., Dice, Recall (sensitivity), SSIM scores, and pixel-wise mean squared error^27–29^ were evaluated for comparing the similarity between the virtual painting images and the ground truth images (see the “Materials and methods” section). The score ‘1’ in the Dice and Recall test means the highest similarity between the ground truth group and the virtual stain group. The lower is the score, the poorer is the similarity. Since most of the pixels in single-channel IF images are in black, these two metrics can robustly reflect both pixel-level and microenvironment-level virtual painting fidelity. We classified each pixel in the generated images into categories of true positive (TP), false positive (FP), and false negative (FN) based on the overlap with the original fluorescent image (GT). A pixel was considered TP if it was present in the GT and its grayscale color intensity differed by no more than 2 levels (0–255) from the GT. As all three models were trained and tested on the same dataset, we conducted a paired Wilcoxon rank-sum test to calculate the *p*-value for the statistical analysis. All the evaluations were implemented in the Python Programming Language. We used scikit-image/skimage for SSIM, SciPy for Dice coefficient, and scikit-learn/sklearn for mean squared error, respectively.

## Data Availability

The raw and pre-processed full-resolution images will be available upon request. A publicly processed version is available at: https://www.dropbox.com/sh/366b1ygood6rvse/AAAAJIQ3OElBqXKbv7OZ7_M6a?dl=0.

## Code availability

All codes that produced the findings of the study are available at: https://github.com/MGH-Gut/PhaseFIT.
